# Incident cardiovascular disease and particulate matter air pollution in South Korea using a population-based and nationwide cohort of 0.2 million adults

**DOI:** 10.1186/s12940-020-00671-1

**Published:** 2020-11-09

**Authors:** Ok-Jin Kim, Soo Hyun Lee, Si-Hyuck Kang, Sun-Young Kim

**Affiliations:** 1 Department of Cancer Control and Population Health, Graduate School of Cancer Science and Policy, National Cancer Center, Goyang-Si, Gyeonggi-Do South Korea; 2 Graduate School of Public Health, Seoul National University, Seoul, South Korea; 3 Cardiovascular Center, Seoul National University Bundang Hospital, Seongnam-si, Gyeonggi-Do South Korea; 4 Department of Internal Medicine, Seoul National University, Seoul, South Korea

**Keywords:** Cardiovascular disease, Fine particle, Incidence, Long-term exposure, Nationwide cohort

## Abstract

**Background:**

While many studies reported the association between long-term exposure to particulate matter air pollution (PM) and cardiovascular disease (CVD), few studies focused on incidence with relatively high-dose exposure using a nationwide cohort. This study aimed to investigate the association between long-term exposure to PM_10_ and PM_2.5_ and incidence of CVD in a nationwide and population-based cohort in South Korea where the annual average concentration of PM_2.5_ is above 20 μg/m^3^.

**Methods:**

We selected 196,167 adults in the National Health Insurance Service-National Sample Cohort (NHIS-NSC) constructed based on the entire South Korean population. Incidence of four CVD subtypes including ischemic heart disease (IHD), myocardial infarction, heart failure, and stroke, and total CVD including all four was identified as the first diagnosis for 2007–2015. To assess individual exposures, we used annually-updated district-level residential addresses and district-specific PM concentrations predicted by a previously developed universal kriging prediction model. We computed individual-level long-term PM concentrations for four exposure windows: previous 1, 3, and 5 year(s) and 5 years before baseline. We applied time-dependent Cox proportional hazards models to estimate hazard ratios (HRs) of incident CVDs per 10 μg/m^3^ increase in PM_10_ and PM_2.5_ after adjusting for individual- and area-level characteristics.

**Results:**

During 1,578,846 person-year, there were 33,580 cases of total incident CVD. Average PM_10_ and PM_2.5_ concentrations for the previous 5 years were 52.3 and 28.1 μg/m^3^, respectively. A 10 μg/m^3^ increase in PM_2.5_ exposed for the previous 5 years was associated with 4 and 10% increases in the incidence of total CVD (95% confidence interval: 0–9%) and IHD (4–16%), respectively. HRs tended to be higher with earlier exposure for IHD and more recent exposure for stroke. The estimated shape of the concentration-response relationship showed non-linear patterns. We did not find evidence of the association for PM_10_.

**Conclusions:**

Using a population-based nationwide cohort exposed to relatively high PM concentration, this study confirmed the association between PM_2.5_ and CVD incidence that was reported in previous studies mostly with low-dose environments. The magnitude and the shape of the association were generally consistent with previous findings.

**Supplementary Information:**

**Supplementary information** accompanies this paper at 10.1186/s12940-020-00671-1.

## Background

Accumulating evidence suggests ambient particulate matter (PM) air pollution as a risk factor for cardiovascular disease (CVD) which is one of the major causes of mortality and public health burden worldwide. Several large cohort studies reported the association between long-term exposure to PM and cardiovascular mortality [1–6]. Recently, increasing numbers of studies focused on CVD incidence [4, 7–18]. However, these studies were mostly based on limited populations such as urban residents and older adults in a few developed countries of North America and Europe and/or a few specific subtypes of CVD.

Uncertainties remain about the magnitude of the association and its variation with high-dose exposure environments. The lack of evidence under high PM concentration has been indicated as a major limitation in completing a dose-response relationship for the full range of the global PM distribution [19–21]. To overcome this limitation, there has been a growing interest in Asian countries where PM air pollution is relatively high. Many Asian countries experienced dramatic and fast economic developments, leading to possibly somewhat different pollution sources, emissions, and chemical composition for PM compared to those under low-dose environments. Along with different socio-demographic characteristics and susceptibility, the pattern and magnitude of resulting health effects could also differ.

A national-scale and population-based cohort with annual updates of extended individual information over more than a decade in South Korea can elucidate the uncertainty in PM-associated disease incidence. The National Health Insurance Service-National Sample Cohort (NHIS-NSC) created from the National Health Insurance Database (NHID) for the entire South Korean population consists of one million people and their individual and biological information including annually updated residential addresses for 2002–2015 [22]. This rich information allows us to assess long-term exposure to PM incorporated with residential mobility and to evaluate the long-term effect of PM on CVD incidence after accounting for other risk factors.

In the present study, taking advantage of the nationwide population-based cohort of a million people with 14 years of follow-ups in South Korea, we attempted to answer important questions for CVD incidence attributable to PM in high-dose environments. Specifically, this study aimed to examine the association between long-term exposure to PM_10_ and PM_2.5_ and incident CVD and to assess their concentration-response relationships. We also investigated the different exposure windows and population characteristics to determine significant exposure period and susceptibility.

## Methods

### Study population

Our study population is based on the NHIS-NSC, an administrative nationwide cohort created from the NHID in South Korea (see Additional File 1: Figure S1) [22]. South Korea established a universal National Health Insurance (NHI) program in 1977 and expanded to the entire population in 2000. Based on the enormous health care information collected, the NHIS established the NHID and created the NHIS-NSC including approximately one million NHI subscribers and medical aid beneficiaries as of 2002 and their follow-up data for 2002–2013. As the second version available since 2017, NHIS-NSC 2.0 includes 14 years of follow-up data for two more years until 2015. The cohort provides district-level home addresses and demographic and socio-economic characteristics updated annually. South Korea is composed of 7 metropolitan cities and 9 provinces at the provincial level in 2007. Each metropolitan city or province includes 5–48 districts with a total of 251–263 districts for 2002–2015 (average size: 429 km^2^ in 2007, range: 3–1818 km^2^) (see Additional file 1: Figure S2) [23, 24]. Basic biological, medical, and health behavior information are also available for a subset of the cohort who underwent the National Health Screening (NHS). The NHS service is offered biannually to employees of all ages and all the other citizens aged 40 years or older. Additional screening service is provided at the ages of 40 and 65 years considered as biologically significant ages over a lifetime.

Among one million total subjects of the NHIS-NSC 2.0, we selected 314,445 people who underwent the NHS for 2005–2007 to utilize CVD-related risk factors such as smoking or underlying diseases. Because the NHIS-NSC was not constructed as a CVD-free cohort, we determined the baseline as January 1, 2007, and selected the people who had not been diagnosed with any circulatory diseases for the 5 years of 2002–2006. This selection also allows relatively long periods of exposure for at least 5 years before CVD events and helps avoid possible outcome misclassification derived by our reliance on diagnostic code for outcome definition. We also applied additional inclusion criteria: 30–84 years old in 2007, as a risk group for CVD commonly used in previous studies; no severe disability grades; and complete information for addresses and all covariates. The final study population included 196,167 adults. None of these subjects were lost during the follow-up period, as our cohort was created based on the NHI which is the universal health care system.

### Long-term exposure assessment

To assess individual long-term exposure to PM for NHIS-NSC subjects, we estimated district-specific annual-average PM_10_ and PM_2.5_ concentrations for 2002–2014 by using two different approaches. For PM_10_, we predicted annual-average concentrations at 83,463 centroids of residential census tracts using a pointwise prediction model for South Korea (see Additional file 1: Figure S3) [25]. This nationwide prediction model was developed in a universal kriging framework including summary predictors of more than 300 geographic variables and spatial correlation based on air quality regulatory monitoring data for 2001–2015 in South Korea. The South Korean regulatory monitoring network included a total of 294 sites in 2010, located in about 60% of districts [26]. The model showed moderate performance with cross-validated R^2^ of 0.45, which is comparable with R^2^s of 0.37–0.62 in the nationwide PM_10_ models in the U.S., Europe, and Asia [27–30]. Using predicted annual-average concentrations at census tract centroids, we computed an average concentration in each district as individual-level exposure, because address information in the NHIS-NSC is limited to the district level for confidentiality [26].

We predicted district-specific annual-average PM_2.5_ concentrations by using ratios of PM_2.5_ to PM_10_ and PM_10_ predictions [31]. The ratio-based approach was frequently applied in previous studies, particularly when PM_2.5_ monitoring data were limited [32–35]. Their predictive ability was relatively high despite the simplicity of the approach. In South Korea, regulatory air quality monitoring data for PM_2.5_ are available only in Seoul and Busan, the first and second-largest metropolitan cities, before 2015 when nationwide data are available, as opposed to PM_10_ data nationally available since 2001. Using the data in Seoul for 2002–2014, we computed the ratio of annual average PM_2.5_ to PM_10_ at each site in Seoul, multiplied by the regional adjustment factor, and then multiplied by predicted district-specific annual-average PM_10_ concentrations in the entire country for 2002–2014. The regional adjustment factor was calculated as the proportion of the ratio in Seoul to the ratio in each of the other metropolitan cities and provinces based on the data in 2015. We applied this factor to account for the difference in the ratio between Seoul and other areas, as the annual ratios for 2001–2014 were computed based on the monitoring data in Seoul. Our validation using PM_2.5_ data in Busan showed good model performance with R^2^ of 0.79.

### Incident cardiovascular disease

The incident CVD cases were defined as the first events of CVD during 2007–2015 among the NHIS-NSC subjects who had never been diagnosed with any circulatory diseases during 2002–2006. In the present study, we focused on four CVD subtypes that were reported for the association with long-term PM in previous studies of CVD incidence: ischemic heart disease (IHD), myocardial infarction (MI), stroke, and heart failure. These four diseases were identified based on the International Classification of Disease 10th revision (ICD-10) code for primary or secondary diagnosis: I20-I25, I21-I23, I60-I64, and I50, respectively. Total CVD included all four CVD events, as often defined in previous cohort studies [4, 6, 36, 37]. For those who were diagnosed with more than one CVD, the earliest diagnosis was included.

### Individual- and area-level covariates

We used various individual- and area-level characteristics obtained from the NHIS-NSC 2.0 and other national survey data as covariates in our health analysis. Individual-level covariates were sex, age, income in decile, insurance type, smoking status, frequency of alcohol use and physical exercise, body mass index (BMI), and preexisting comorbidity for diabetes, hyperlipidemia, and hypertension at baseline. These covariates were commonly included as confounders in previous cohort studies of PM and CVD [4, 14, 15, 17, 36]. Preexisting comorbidity was identified based on blood tests and physical examination results in the NHS: fasting blood sugar level > 126 mg/dL for diabetes [38], total cholesterol level > 240 mg/dL for hyperlipidemia [39], and diastolic blood pressure > 140 mmHg or systolic blood pressure > 90 mmHg for hypertension [40]. We also included three area-level covariates that represent the socio-economic characteristics of residential districts at baseline in 2007. Three area-level covariates were district-specific percentages of elderly people (≥ 65 years) and high school graduates, and gross regional domestic product (GRDP). The proportion of the elderly was often used as an indicator of area-level socioeconomic status in South Korea based on high poverty rate and low economic activity of the elderly population [41, 42]. Proportions of elderly people and high school graduates were obtained from the 2000 Census [43], whereas GRDP was from general national statistics in 2005 [44]. We converted some individual-level covariates to categorical variables to represent non-linear relationships with CVD: young or older adults (30–64 or 65–84 years), four income groups (0–20, 20–50 50–80, or 80–100%), and non-obese or obese people (BMI < 25 or ≥ 25 kg/m^2^). We also categorized three area-level covariates into their quantiles (very low, low, high, or very high) based on the distribution across about 250 districts. Age was included as continuous and binary (30–64 vs. 65–84 years) variables.

### Statistical analysis

We used time-dependent Cox proportional hazards model with the time window of one year, and estimated hazard ratios (HRs) and 95% confidence intervals (CIs) of incidence of total CVD and four subtypes per 10 μg/m^3^ increase in long-term PM_10_ and PM_2.5_ concentrations. We chose study-on-time as our time scale to account for the relationship between air pollution and time, as commonly applied in previous epidemiological studies of air pollution [11, 13, 45]. We assessed the long-term exposure of each subject for the previous 5 years by incorporating residential mobility and using predicted district-specific annual-average concentrations of PM_10_ and PM_2.5_. We also applied two additional exposure periods as more recent exposure for the previous 1 and 3 years. In addition, as more early exposure, we employed the 5-years average for 2002–2006 before the baseline and applied time-constant Cox model. Separate analyses were performed by four exposure periods. All covariates except PM_10_ and PM_2.5_ were treated as being constant over time because biological and individual characteristics obtained from the NHS were irregularly available depending on subjects’ participation in the NHS. The survival time of each subject was calculated from January 1, 2007, to the earliest date of CVD event, death, drop-out, or the end of follow-up on December 31, 2015.

We applied five health analysis models including progressively adjusted covariates. In model 1, we included sex, age, and long-term PM concentration. We added individual-level covariates such as income percentile, insurance type, smoking status, frequency of alcohol consumption and exercise, and obesity in model 2. Model 3 additionally included three preexisting diseases of hypertension, diabetes, and hyperlipidemia. In models 4 and 5, we added all three area-level variables to models 2 and 3. Model 5 was considered as our primary model. In addition, using the primary health analysis model and PM exposure for the previous 5 years, we explored the shape of the concentration-response (C-R) relationship between long-term exposure to PM and incident CVD [46].

To investigate susceptible subpopulation in the association, we performed stratified analyses by individual-level characteristics, region, and area type. The region was classified into two categories of 7 metropolitan cities and 9 provinces, while the area type included urban, suburban, and rural areas [47]. As our focus lies in the identification of susceptible population subgroups more than the examination of the difference between subgroups, we investigated the association in each subgroup using stratified analysis instead of interaction analysis. However, we also determined the difference relying on the non-overlapping 95% CIs between subgroups [48].

For sensitivity analyses, we redefined the study area and incident CVD cases. Our exposure assessment relying on district averages may produce exposure measurement error in the national analysis because the area size of districts substantially varies across metropolitan cities and provinces (median size = 35 to 580 km^2^ in 2007) (see Additional file 1: Figure S4) [23]. To assess this measurement error, we restricted our study area to the Seoul Metropolitan Area (SMA) including Seoul and its neighboring metropolitan city and province, Incheon and Gyeonggi-do, and compared to our primary findings in the national analysis. As the SMA consists of half of the South Korean population and 66 districts with relatively small sizes (median size = 44 km^2^ in 2007) [23], this restricted analysis could allow us to avoid exposure measurement error resulting from assigning the same district-specific exposure to all the people living in a large district. Because the characteristics of districts in the SMA was relatively homogeneous, we did not adjust for area-level variables in our health analysis. Second, we restricted incident cases to CVD hospitalization by excluding moderate CVD cases. Together with our application of the 5-year CVD-free period, this restriction can help avoid possible misclassification driven by using medical claim data. Lastly, we added CVD deaths without previous CVD diagnoses to incident cases to include fatal CVD. All statistical analyses were carried out in SAS Enterprise Guide (version 7.13; SAS Institute Inc., Cary, NC, USA) and R (version 3.6.1, R Core Team 2014, Vienna, Austria).

## Results

Table 1 shows the summary statistics of individual- and area-level characteristics at baseline for 196,167 NHIS-NSC 2.0 subjects. The study population was predominantly middle-aged (mean: 46.6 years; standard deviation (SD): 11.0) and never smokers (67.7%). Almost half of the people (45.3%) lived in metropolitan cities. Current smokers and frequent alcohol users (≥ 3 times/week) comprised 23.1 and 10.3%, respectively. The prevalence of hypertension, diabetes, and hyperlipidemia at baseline was 21.0, 6.4, and 12.0%, respectively. More than 70% of people lived in the districts where GRDP and the proportion of high school graduates were greater than the district median. A total of 33,580 subjects had one or more incident cardiovascular events during 1,578,846 person-years. Incidence of total CVD, IHD, MI, stroke, and heart failure were 21.3, 13.1, 0.9, 6.5, and 1.9 per 10^3^ person-years, respectively.

**Table 1:** Descriptive statistics of individual- and area-level characteristics of 196,167 subjects of the National Health Insurance Service-National Sample Cohort 2.0 in South Korea for 2007–2015

Characteristics		Total (*n* = 196,167)
Sex (%)	Male	53.5
Average age (years, mean ± SD)		46.6 ± 11.0
Age (years, %)	30–64	92.1
Income percentile ^a^ (%)	0–20%	11.8
	20–50%	24.6
	50–80%	36.9
	80–100%	26.7
Insurance type (%)	Self-employed	40.3
	Employee	59.7
Smoking status (%)	Never	67.7
	Former	9.2
	Current	23.1
Alcohol use (%)	≥ 3 times/week	10.3
Physical activity (%)	≥ 3 times/week	20.4
BMI (%)	≥ 25 kg/m^2^	33.4
Comorbidity (%)	Hypertension	21.0
	Diabetes	6.4
	Hyperlipidemia	12.0
Region (%)	Metropolitan cities	45.3
	Provinces	54.7
Gross Regional Domestic Products^b^ (%)	Very low	8.0
	Low	17.7
	High	36.0
	Very High	38.3
Percent of the high school graduated or more^c^ (%)	Very low	9.9
	Low	15.9
	High	34.4
	Very High	39.9
Percent of the elderly^d^ (%)	Very low	37.6
	Low	35.9
	High	16.1
	Very High	10.3
Cardiovascular disease (n, incidence per 10^3^ person-year)	Total	33,580 (21.3)
	Ischemic heart disease	20,604 (13.1)
	Myocardial infarction	1367 (0.9)
	Stroke	10,201 (6.5)
	Heart Failure	3033 (1.9)

Abbreviations: *SD* standard deviation; *BMI* body mass index

^a^ NHIS-NSC provided income as percentiles (“The manual for User of the National Health Insurance Service of National Sample Cohort Database”)

^b^ Gross regional domestic products were categorized as very low (< 1110 M USD), low (1110–2600 M USD), high (2600–8354 M USD), and very high (≥ 8354 M USD) based on the distribution across approximately 250 districts

^c^ Percent of high school graduates was categorized as very low (< 34.3%), low (34.3–46.6%), high (46.6–53.2%), and very high (≥ 53.2%) based on the distribution across approximately 250 districts

^d^ Percent of the elderly aged 65 years or more was categorized as very low (< 5.4%), low (5.4–8.2%), high (8.2–14.9%), and very high (≥14.9%) based on the distribution across approximately 250 districts

The mean of individual-level long-term PM_10_ and PM_2.5_ concentrations of 196,167 subjects of the NHIS-NSC 2.0 for the previous 5 years were 52.3 and 28.1 μg/m^3^ (SD: 6.2 and 3.6), respectively (Table 2). Average long-term PM concentrations varied across four different exposure periods. Compared to the concentrations for relatively long periods for the previous 3 or 5 years, PM_10_ and PM_2.5_ concentrations for the previous 1 year showed slightly lower means (50.5 and 26.4 μg/m^3^). The early exposure for 2002–2006 showed highest means (55.7 and 31.2 μg/m^3^).

**Table 2:** Descriptive statistics of long-term PM_10_ and PM_2.5_ concentrations (μg/m^3^) of 196,167 subjects of the National Health Insurance Service-National Sample Cohort 2.0 in South Korea for 2007–2015 by four exposure periods

Pollutants	Exposure period	Mean ± SD	Interquartile range	Range
PM_10_	Previous 1 year	50.5 ± 6.8	9.2	33.6–76.0
	Previous 3 years	51.2 ± 6.4	10.1	33.8–74.2
	Previous 5 years	52.3 ± 6.2	9.7	36.6–74.2
	5 years before baseline^a^	55.7 ± 6.6	11.4	38.4–71.4
PM_2.5_	Previous 1 year	26.4 ± 3.1	4.2	18.0–38.1
	Previous 3 years	27.5 ± 3.9	5.1	18.8–40.8
	Previous 5 years	28.1 ± 3.6	5.5	19.0–39.8
	5 years before baseline^a^	31.2 ± 3.4	4.5	21.2–39.8

Abbreviations: *PM*_*10*_ particulate matter 10 μm or less in diameter. *PM*_*2.5*_ particulate matter 2.5 μm or less in diameter. *SD* standard deviation

^a^5 years before baseline, 5-year average PM concentration for 2002–2006 before the baseline in 2007

Table 3 shows HRs and 95% CIs of incidence of total CVD and four subtypes for individual-level concentrations of PM_10_ and PM_2.5_ over the previous 5 years. Using our primary model, model 5, we found a marginal association between PM_2.5_ and total incident CVDs after adjusting for individual- and area-level characteristics (HR: 1.04 (95% CI: 1.00,1.09)). The effect estimates were higher for IHD and MI, showing 10 and 11% increases for a 10 μg/m^3^ increase in PM_2.5_ (95% CI: 1.04,1.16 and 0.90,1.37, respectively), although the association for MI was statistically non-significant. Different from three subtypes, heart failure showed a significantly negative association. HRs were generally higher in models 4 and 5, where area-level confounders were additionally adjusted, compared to those in model 3 including individual confounders only. The estimated C-R relationship for PM_2.5_ showed non-linear patterns with total CVD and IHD. HRs were close to 1 below 30 μg/m^3^ and modestly increased until approaching the plateau at about 40 μg/m^3^ (see Additional file 1: Figure S5, Figure S6). For PM_10_, we did not find any associations for total CVD (HR:1.00 (95% CI: 0.98,1. 02)) and four subtypes.

**Table 3:** Adjusted hazard ratios and 95% confidence intervals of incident cardiovascular diseases for a 10 μg/m^3^ increase in long-term PM_10_ and PM_2.5_ concentrations for the previous 5 years by five health analysis models in 196,167 subjects of the National Health Insurance Service-National Sample Cohort 2.0 in South Korea for 2007–2015

Pollutants	Cardiovascular event	Hazard ratio (95% confidence interval)				
		Model 1^a^	Model 2^b^	Model 3^c^	Model 4^d^	Model5^e^
PM_10_	Total	0.97 (0.95,0.99)	0.97 (0.96,0.99)	0.96 (0.94,0.98)	1.00 (0.98,1.03)	1.00 (0.98,1.02)
	Ischemic heart disease	1.01 (0.98,1.03)	1.00 (0.97,1.03)	1.00 (0.97,1.02)	1.01 (0.98,1.03)	1.00 (0.97,1.03)
	Myocardial Infarction	0.95 (0.86,1.05)	0.95 (0.86,1.05)	0.94 (0.85,1.04)	0.97 (0.87,1.09)	0.96 (0.86,1.08)
	Stroke	0.94 (0.90,0.97)	0.94 (0.91,0.98)	0.94 (0.91,0.98)	1.01 (0.98,1.04)	1.01 (0.97,1.05)
	Heart Failure	0.84 (0.78,0.90)	0.84 (0.79,0.90)	0.84 (0.78,0.90)	0.96 (0.89,1.04)	0.96 (0.89,1.04)
PM_2.5_	Total	1.00 (0.96,1.04)	1.00 (0.97,1.04)	1.00 (0.97,1.04)	1.04 (1.00,1.09)	1.04 (1.00,1.09)
	Ischemic heart disease	1.10 (1.05,1.16)	1.10 (1.05,1.15)	1.10 (1.05,1.15)	1.10 (1.05,1.15)	1.10 (1.04,1.16)
	Myocardial Infarction	1.06 (0.88,1.28)	1.06 (0.88,1.28)	1.05 (0.87,1.27)	1.11 (0.90,1.37)	1.11 (0.90,1.37)
	Stroke	0.92 (0.86,0.99)	0.94 (0.87,1.00)	0.93 (0.87,1.00)	1.01 (0.94,1.09)	1.01 (0.94,1.09)
	Heart Failure	0.69 (0.61,0.79)	0.71 (0.62,0.80)	0.71 (0.62,0.81)	0.84 (0.73,0.96)	0.84 (0.73,0.96)

Abbreviations: *PM*_*10*_ particulate matter 10 μm or less in diameter. *PM*_*2.5*_ particulate matter 2.5 μm or less in diameter. Total included four subtypes of cardiovascular diseases: ischemic heart disease, myocardial infarction, stroke, and heart failure

^a^ Model 1: PM_10_ or PM_2.5_, sex, and age

^b^ Model 2: Model 1 + income, smoking, alcohol use, obese, and physical activity

^c^ Model 3: Model 2 + comorbidity of hypertension, diabetes, or hyperlipidemia

^d^ Model 4: Model 2 + area-level gross regional domestic products, percent of high school graduated or more, and percent of the elderly

^e^ Model 5: Model 3 + area-level gross regional domestic products, percent of high school graduated or more, and percent of the elderly

**Fig. 1:**
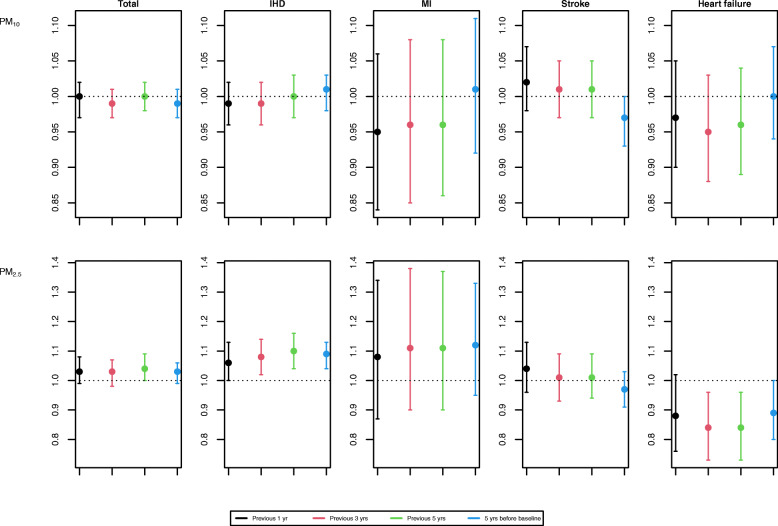
Adjusted hazard ratios and 95% confidence intervals of incident cardiovascular diseases for a 10 μg/m^3^ increase in long-term PM_10_ and PM_2.5_ concentrations by four exposure windows

**Fig. 2:**
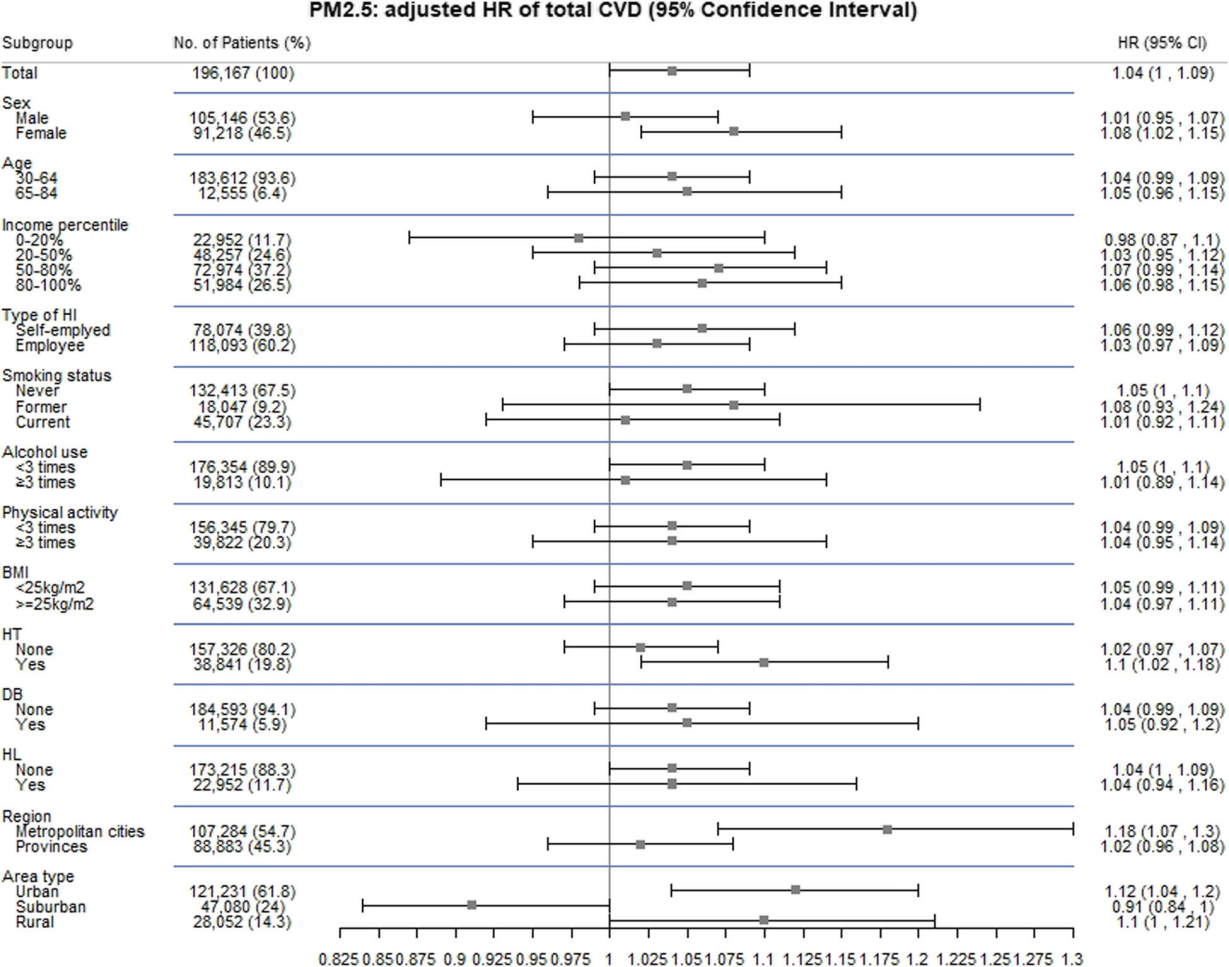
Adjusted hazard ratios and 95% confidence intervals of total incident cardiovascular diseases for a 10 μg/m^3^ increase in long-term PM_2.5_ concentrations for the previous 5 years stratified by sex, age, income, insurance type, smoking status, alcohol use, physical activity, obese, co-morbidity of hypertension, diabetes, and hyperlipidemia, region, or area type

In the comparison to recent exposure for the previous 1 or 3 years and early exposure for 2002–2006, HRs for total CVD were consistent with that using the exposure for the previous 5 years (Fig. 1). However, CVD subtypes showed somewhat different patterns: higher HRs for early exposure with IHD and MI and for recent exposure with stroke. Figure 2 shows subgroup analyses for the association between PM_2.5_ for the previous 5 years and incident total CVD by individual and areal characteristics. We found significantly positive associations for the people who were female, hypertensive patients, and residents in metropolitan cities or urban areas, while marginal associations were observed for never smokers, light alcohol drinkers, hyperlipidaemic patients, and rural residents.

When we restricted the study area to the SMA, findings were generally consistent with the primary findings for the entire country. Effect estimates increased particularly for PM_10_ and stroke and for both PM and heart failure but became mostly statistically non-significant, possibly because of the reduced power (see Additional file 1: Table S1). When we redefined our incident cases to hospital admissions, effect estimates dramatically decreased (see Additional file 1: Table S2, Figure S7). Defining incident CVDs based on CVD diagnosis as well as CVD death did not alter our findings of the primary analysis using CVD diagnosis only (see Additional file 1: Table S3, Figure S8).

## Discussion

Using the population-based nationwide cohort of about 0.2 million adults over 9 years in South Korea, this study investigated the association between long-term exposure to PM and incident cardiovascular diseases. This cohort particularly strengthened our investigation based on annually-updated address information and an extended set of individual- and area-level confounders for the people who were sampled from the entire South Korean population and exposed to the high level of PM greater than 50 and 25 μg/m^3^ of annual average PM_10_ and PM_2.5_ concentrations, respectively. Our results showed the positive association of the incidence of total CVD and IHD with long-term exposure to PM_2.5_. The shape of this association was non-linear. The exposure period related to increased risk estimates tended to vary by CVD subtypes with early exposure for stroke and delayed exposure for IHD and MI. Potential susceptible populations were females, urban residents, and patients with hypertension.

As one of a few nationwide population-based studies of the association between long-term exposure to PM and incidence of CVD in Asia, we provided evidence of the association with PM_2.5_ but not with PM_10_. To compare our results with previous findings, we summarized large cohort studies of PM and CVD incidence in Table S4. Although many studies of long-term PM_2.5_ or PM_10_ reported positive associations with CVD mortality [18, 36, 49], studies focusing on CVD incidence showed inconsistent results between PM_2.5_ and PM_10_, as shown in our study. Effect estimates of PM_10_ were generally positive but statistically non-significant [4, 6, 7, 12, 50], with some studies of significantly negative effect estimates [12, 13, 50]. A recent systematic review also reported the association with both CVD mortality and incidence for PM_2.5_ but only with CVD mortality for PM_10_ [51]. These findings of the stronger association for PM_2.5_ compared to PM_10_ could be explained by biological plausibility based on the smaller size of PM_2.5_ [52] and/or reduced exposure misclassification owing to high prediction ability of individual-level exposure [53]. In addition, while PM concentration is higher in South Korea than in North America and Europe, the magnitude of our effect estimates was similar to those under low concentration environments. This suggests a non-linear C-R relationship with consistent risks above a relatively low exposure level.

Our analysis focusing on the SMA generally showed consistent findings with increased effect estimates compared to those in the nationwide analysis. Most U.S. studies that showed positive associations were predominantly based on urban, white, and/or female populations [4, 14, 36]. In contrast, a few population-based cohort studies reported null or negative associations as shown in our results [7, 10, 12]. Likewise, when we restricted our study area to the SMA, the PM_10_ effect estimates which were close to 1 or negative in the national analysis became positive for total and subtype CVD. This difference could be in part due to lower CVD incidence and higher PM concentration in the SMA that is mostly made up of urban areas and the reverse pattern in the non-SMA largely composed of suburban and rural areas (see Additional file 1: Table S5). In particular, the incidence of heart failure in the non-SMA was twice as many as that in the SMA: this much higher incidence, compared to other CVD subtypes, along with low exposure in the non-SMA possibly resulted in the negative association with PM_2.5_ in the nationwide analysis. Although we aggressively adjusted for individual and area-level confounders, these may not be sufficient to account for different characteristics across regions. It is also possible that the findings in the non-SMA were affected by exposure misclassification resulting from the application of district-average exposure to large rural districts.

Our results of the negative association using hospitalization may indicate a unique feature of hospitalization rather than a protective effect. Our sensitivity analysis that restricted incident CVD to hospitalization showed a substantial decrease in effect estimates with significantly negative effect estimates of total CVD, IHD, and stroke (see Additional file 1: Table S2, Figure S7). Some previous studies based on medical records or claim data used hospital admission to reduce the possibility of outcome misclassification [10, 11, 54]. However, when we restricted to the people who had been hospitalized for CVD, they tended to live more in suburban or rural areas and be exposed to lower PM concentration compared to the total population (see Additional file 1: Table S6). Although this population was older and had less healthy behaviors indicating possible susceptibility to the CVD risk of PM, their lower exposure or exposure misclassification might have played a more important role resulting in the negative association.

Identifying a critical period of exposure to PM associated with CVD incidence can provide practical guidance to avoid the adverse cardiovascular effect of air pollution. In our results using the four exposure periods from the previous 1 year to the 5 years before baseline, effect estimates were generally consistent for total CVD but varied slightly across subtypes. Higher effect estimates of stroke for the previous 1 to 5 years of exposure indicate relatively acute response to PM. On the contrary, IHD and MI showed increased effect estimates as the exposure period was extended up to the 5 years before baseline. At least four previous studies compared the effect estimates of long-term PM on CVD mortality or morbidity across various exposure periods [6, 13, 14, 36]. These studies were all based on the Nurses’ Health Study cohort that updated address information biennially and investigated different exposure windows from the previous 1 to 120 months. In their findings, PM exposures for the previous 12 to 48 months gave positive effect estimates whereas there were no or negative associations with more recent or distant exposures. The high contribution of relatively recent exposure compared to early exposure consistently found in the Nurses’ Health Study and our cohort, especially for stroke, indicates a possible underestimation of risk for CVD incidence using baseline exposure that was commonly applied in many previous studies [4, 7–12, 15, 37, 50, 55].

In our subgroup analysis, we observed the association in females, patients with hypertension, and urban residents. These high-risk subgroups were also found in previous studies of PM and CVD. U.S. studies using female cohorts consistently reported the association between long-term PM exposure and incident CVD [4, 6, 14, 36]. A study that focused on gender difference showed a greater risk of fatal coronary heart disease in females than males [9]. Most Northern American cohort studies that reported the association were conducted in metropolitan urban areas [4, 6, 9, 14, 36], whereas some population-based or nationwide studies reported negative results [7, 10, 12]. As hypertension is a well-known risk factor for CVD [56], the adverse effect of PM on CVD could be larger in hypertensive patients. In addition, there was a marginal association among never smokers, light alcohol drinkers, and rural residents. High risk of PM in never smokers may be due to the absence of other strong risk factors of CVD such as smoking [15]. We found significantly and marginally positive associations in urban and rural districts, respectively, while there was a marginally negative association in suburban districts. While high risk in the urban population can be explained by high exposure, high risk in the rural population exposed to relatively low PM air pollution could be related to high susceptibility [57]. Interestingly, although exposure to PM was lower both in rural and suburban districts than urban districts, the population in rural areas tended to be older, had lower socioeconomic status, and engaged more with less healthy behaviors (see Additional file 1: Table S7). However, we should be careful to interpret our findings in rural areas because of possible exposure misclassification.

The present study includes some limitations to address for future research. First, we used district-specific exposure to assess individual-level exposure because address information of the NHIS-NSC is available at the district level. This relatively coarse spatial scale of exposure assessment might have affected exposure misclassification and bias in health effect estimation. However, because PM is considered as a regional pollutant that is relatively homogeneous in a large area, the impact could be relatively small. Future studies should confirm our findings based on fine spatial-scale address data. Second, we used a simple approach relying on the ratio of PM_2.5_ to PM_10_ to estimate long-term PM_2.5_ concentrations given limited monitoring data before 2015. Future studies should use new data sources and/or more refined modeling approaches to overcome the data limitation. Third, we defined CVD incidence relying on the diagnosis code. However, we attempted to reduce the possibility of misclassification by applying a conservative inclusion criterion that restricted the study population to those never diagnosed with CVD for a relatively long-term period of 5 years. Finally, we did not apply two- or multi-pollutant models including other criteria pollutants, because of a concern about exposure misclassification and/or unavailability of predictions at the national scale. Application of district-average exposure given the limited availability of NHIS-NSC address data could result in large exposure misclassification for local pollutants such as NO_2_ as opposed to PM indicated as a regional pollutant [58]. The national prediction model was not available for the other gaseous criteria pollutants. Future studies should develop national prediction models for other pollutants and confirm our findings after accounting for their impacts.

Despite these limitations, our study has several strengths. Our large population-based nationwide cohort sampled from the entire South Korean population and followed up for about 10 years allowed us to provide generalizable evidence of the association between the prolonged exposure to PM_2.5_ and the incidence of CVD. Extended information on various socio-demographic and biological characteristics helped reassure the association after accounting for potential confounders and investigate susceptible populations, while annually-updated addresses enabled the improvement of accuracy in exposure assessment.

## Conclusions

To reach a consensus on the health effect of PM and the burden of disease on a global scale, the contribution of local studies is highly important. Focusing on cardiovascular disease incidence in a population-based and nationwide cohort and using local exposure assessment for PM in South Korea, our study confirmed the association between long-term exposure to fine particulate matter air pollution and cardiovascular diseases.

## Supplementary Information

**Additional file 1: Table S1.** Adjusted hazard ratios and 95% confidence intervals of incident cardiovascular diseases for a 10 μg/m^3^ increase in long-term PM_10_ and PM_2.5_ concentrations for the previous 5 years by three health analysis models in 84,412 Seoul Metropolitan Area residents of the National Health Insurance Service-National Sample Cohort 2.0, South Korea, for 2007–2015. **Table S2.** Adjusted hazard ratios and 95% confidence intervals of incidence of total cardiovascular diseases and four subtypes defined as hospital admissions for a 10 μg/m^3^ increase in long-term PM_10_ and PM_2.5_ concentrations for the previous 5 years by five health analysis models in 196,167 subjects of the National Health Insurance Service-National Sample Cohort 2.0 in South Korea for 2007–2015. **Table S3.** Adjusted hazard ratios and 95% confidence intervals of incident cardiovascular diseases including cardiovascular deaths for a 10 μg/m^3^ increase in long-term PM_10_ and PM_2.5_ concentrations for the previous 5 years by five health analysis models in 196,167 subjects of the National Health Insurance Service-National Sample Cohort 2.0 in South Korea for 2007–2015. **Table S4**. Previous large cohort studies of the association between long-term exposure to PM_10_ or PM_2.5_ and incident cardiovascular diseases. **Table S5.** Summary statistics of individual-level long-term concentration of PM_10_ and PM_2.5_ across four exposure periods and the number of events and incidence of total and subtypes of cardiovascular diseases in 196,167 subjects of the National Health Insurance Service-National Sample Cohort 2.0 in South Korea for 2007–2015 by the Seoul Metropolitan Area (SMA) and non-SMA. **Table S6.** Descriptive summary statistics of individual- and area-level characteristics, and long-term PM_10_ and PM_2.5_ concentrations of 196,167 total subjects of the National Health Insurance Service-National Sample Cohort 2.0 in South Korea for 2007–2015 by diagnosis and hospitalization of cardiovascular diseases. **Table S7.** Descriptive summary statistics of individual- and area-level characteristics, and long-term PM_10_ and PM_2.5_ concentrations of 196,167 total subjects of the National Health Insurance Service-National Sample Cohort 2.0 in South Korea for 2007–2015 by diagnosis and hospitalization of cardiovascular diseases. **Figure S1.** Schematic diagram of subject exclusion criteria and numbers of the National Health Insurance Service- National Sample Cohort 2.0 subjects included or excluded after the application of the criteria. **Figure S2**. Map of 7 metropolitan cities and 9 provinces in South Korea, 2010. **Figure S3.** Maps of predicted annual- average concentrations of PM_10_ across 245,248, and 252 districts in South Korea by 2002, 2007, and 2014, respectively (modified from the maps on https://tabsoft.co/2T7v6ti). **Figure S4**. Box plot of the area sizes of districts by 7 metropolitan cities and 9 provinces, South Korea, in 2007. **Figure S5**. The concentration-response relationship between individual-level long-term concentration of PM_2.5_ for the previous 5 years and the incidence of total cardiovascular disease in 196,167 subjects of the National Health Insurance Service-National Sample Cohort 2.0 in South Korea for 2007–2015. **Figure S6**. The concentration-response relationship between individual-level long-term PM_2.5_ concentration for the previous 5 years and the incidence of ischemic heart disease in 196,167 subjects of the National Health Insurance Service-National Sample Cohort 2.0 in South Korea for 2007–2015. **Figure S7.** Adjusted hazard ratios and 95% confidence intervals of incidence of total cardiovascular diseases and four subtypes defined as hospital admissions for a 10 μg/m^3^ increase in long-term PM_10_ and PM_2.5_ concentrations by four exposure periods. Hazard ratios were adjusted for sex, age, income, smoking, alcohol use, obesity, physical activity, comorbidity (hypertension, diabetes, or hyperlipidemia), area-level gross regional domestic products, percent of high school graduated or more, and percent of the elderly. HR, hazard ratio; CI, confidence interval; PM_10_, particulate matter 10 μm or less in diameter; PM_2.5_, particulate matter 2.5 μm or less in diameter; IHD, ischemic heart disease; MI, myocardial infarction. **Figure S8**. Adjusted hazard ratios and 95% confidence intervals of incident cardiovascular diseases including death from cardiovascular diseases for a 10 μg/m^3^ increase in long-term PM_10_ and PM_2.5_ concentrations by four exposure windows. Hazard ratios were adjusted for sex, age, income, smoking, alcohol use, obesity, physical activity, comorbidity (hypertension, diabetes, or hyperlipidemia), area-level gross regional domestic products, percent of high school graduated or more, and percent of the elderly. HR, hazard ratio; CI, confidence interval; PM_10_, particulate matter 10 μm or less in diameter; PM_2.5_, particulate matter 2.5 μm or less in diameter; IHD, ischemic heart disease; MI, myocardial infarction.

## Data Availability

The datasets analyzed in this study are available from the corresponding author SY Kim (sykim@ncc.re.kr) on reasonable request.

## Abbreviations

PM: Particulate matter air pollution
CVD: Cardiovascular disease
PM_10_: Particulate matter with a diameter equal to or less than 10 μm
PM_2.5_: Particulate matter with a diameter equal to or less than 2.5 μm
NHIS: National Health Insurance Service
NHIS-NSC: National Health Insurance Service-National Sample Cohort
NHID: National Health Insurance Database
NHI: National Health Insurance
NHS: National Health Screening
IHD: Ischemic heart disease
MI: Myocardial infarction
HR: Hazard ratio
CI: Confidence interval
ICD-10: International Classification of Disease 10th revision
BMI: Body mass index
GRDP: Gross regional domestic product
C-R: Concentration-response
SMA: Seoul Metropolitan Area
SD: Standard deviation
