# Supplementary material for: Incident cardiovascular disease and particulate matter air pollution in South Korea using a population-based and nationwide cohort of 0.2 million adults
Source: Environ Health. 2020 Nov 9;19:113. doi: 10.1186/s12940-020-00671-1 (PMC7653702; doi:10.1186/s12940-020-00671-1)
Supplement: Supplementary file 1 — Additional file 1: Table S1. Adjusted hazard ratios and 95% confidence intervals of incident cardiovascular diseases for a 10 μg/m3 increase in long-term PM10 and PM2.5 concentrations for the previous 5 years by three health analysis models in 84,412 Seoul Metropolitan Area residents of the National Health Insurance Service-National Sample Cohort 2.0, South Korea, for 2007–2015. Table S2. Adjusted hazard ratios and 95% confidence intervals of incidence of total cardiovascular diseases and four subtypes defined as hospital admissions for a 10 μg/m3 increase in long-term PM10 and PM2.5 concentrations for the previous 5 years by five health analysis models in 196,167 subjects of the National Health Insurance Service-National Sample Cohort 2.0 in South Korea for 2007–2015. Table S3. Adjusted hazard ratios and 95% confidence intervals of incident cardiovascular diseases including cardiovascular deaths for a 10 μg/m3 increase in long-term PM10 and PM2.5 concentrations for the previous 5 years by five health analysis models in 196,167 subjects of the National Health Insurance Service-National Sample Cohort 2.0 in South Korea for 2007–2015. Table S4. Previous large cohort studies of the association between long-term exposure to PM10 or PM2.5 and incident cardiovascular diseases. Table S5. Summary statistics of individual-level long-term concentration of PM10 and PM2.5 across four exposure periods and the number of events and incidence of total and subtypes of cardiovascular diseases in 196,167 subjects of the National Health Insurance Service-National Sample Cohort 2.0 in South Korea for 2007–2015 by the Seoul Metropolitan Area (SMA) and non-SMA. Table S6. Descriptive summary statistics of individual- and area-level characteristics, and long-term PM10 and PM2.5 concentrations of 196,167 total subjects of the National Health Insurance Service-National Sample Cohort 2.0 in South Korea for 2007–2015 by diagnosis and hospitalization of cardiovascular diseases. Table S7. Descrip [file 12940_2020_671_MOESM1_ESM.docx]

**Additional file 1**

**Table S1.** Adjusted hazard ratios and 95% confidence intervals of incident cardiovascular diseases for a 10 μg/m^3^ increase in long-term PM_10_ and PM_2.5_ concentrations for the previous 5 years by three health analysis models in 84,412 Seoul Metropolitan Area residents of the National Health Insurance Service-National Sample Cohort 2.0, South Korea, for 2007-2015.

| Pollutant | Cardiovascular event | Hazard ratio (95% confidence interval) | | |
| --- | --- | --- | --- | --- |
|  |  | Model 1^a^ | Model 2^b^ | Model 3^c^ |
| PM_10_ | Total | 1.05 (0.99,1.11) | 1.04 (0.98,1.09) | 1.03 (0.98,1.09) |
|  | Ischemic heart disease | 0.99 (0.93,1.06) | 0.99 (0.92,1.06) | 0.99 (0.92,1.05) |
|  | Myocardial Infarction | 1.05 (0,81,1.36) | 1.03 (0.79,1.35) | 1.03 (0.79,1.34) |
|  | Stroke | 1.14 (1.04,1.26) | 1.12 (1.02,1.23) | 1.12 (1.01,1.23) |
|  | Heart Failure | 1.19 (0.99,1.43) | 1.14 (0.95,1.37) | 1.14 (0.95,1.37) |
| PM_2.5_ | Total | 1.06 (0.95,1.20) | 1.04 (0.93,1.18) | 1.04 (0.93,1.17) |
|  | Ischemic heart disease | 1.11 (0.96,1.29) | 1.10 (0.95,1.27) | 1.09 (0.94,1.27) |
|  | Myocardial Infarction | 1.28 (0.72,2.29) | 1.26 (0.71,2.25) | 1.25 (0.70,2.23) |
|  | Stroke | 0.93 (0.75,1.16) | 0.91 (0.73,1.13) | 0.91 (0.73,1.13) |
|  | Heart Failure | 1.30 (0.85,1.98) | 1.24 (0.81,1.89) | 1.23 (0.81,1.88) |

Abbreviations: PM_10_, particulate matter 10 micrometers or less in diameter. PM_2.5_, particulate matter 2.5 micrometers or less in diameter.

^a^ Model 1: PM_10_ or PM_2.5_, sex, and age

^b^ Model 2: Model 1 + income, smoking, alcohol use, obese, and physical activity

^c^ Model 3: Model 2 + comorbidity of hypertension, diabetes, or hyperlipidemia

**Table S2.** Adjusted hazard ratios and 95% confidence intervals of incidence of total cardiovascular diseases and four subtypes defined as hospital admissions for a 10 μg/m^3^ increase in long-term PM_10_ and PM_2.5_ concentrations for the previous 5 years by five health analysis models in 196,167 subjects of the National Health Insurance Service-National Sample Cohort 2.0 in South Korea for 2007-2015.

| Pollutant | Cardiovascular event | Number of events  (incidence per 10^3^ PY) | Hazard ratio (95% confidence interval) | | | | |
| --- | --- | --- | --- | --- | --- | --- | --- |
|  |  |  | Model 1^a^ | Model 2^b^ | Model 3^c^ | Model 4^d^ | Model5^e^ |
| PM_10_ | Total | 6,131 (3.9) | 0.84 (0.80,0.88) | 0.84 (0.80,0.88) | 0.83 (0.79,0.87) | 0.86 (0.81,0.90) | 0.85 (0.81,0.90) |
|  | Ischemic heart disease | 2,583 (1.6) | 0.78 (0.72,0.84) | 0.81 (0.75,0.87) | 0.81 (0.75,0.87) | 0.80 (074,0.87) | 0.80 (0.73,0.87) |
|  | Myocardial Infarction | 716 (0.5) | 0.95 (0.82,1.09) | 0.94 (0.82,1.08) | 0.93 (0.81,1.07) | 0.92 (0.79,1.08) | 0.91 (0.78,1.07) |
|  | Stroke | 3,278 (2.1) | 0.86 (0.81,0.92) | 0.87 (0.82,0.93) | 0.86 (0.81,0.92) | 0.90 (0.83,0.97) | 0.89 (0.83,0.96) |
|  | Heart Failure | 374 (0.2) | 0.80 (0.66,0.98) | 0.83 (0.68,1.01) | 0.83 (0.68,1.00) | 0.94 (0.75,1.18) | 0.94 (0.75,1.18) |
| PM_2.5_ | Total | 6,131 (3.9) | 0.92 (0.84,1.00) | 0.94 (0.85,1.02) | 0.93 (0.85,1.04) | 0.94 (0.85,1.04) | 0.94 (0.85,1.04) |
|  | Ischemic heart disease | 2,583 (1.6) | 0.97 (0.84,1.12) | 0.98 (0.85, 1.13) | 0.97 (0.84,1.12) | 0.98 (0.84,1.15) | 0.98 (0.84,1.14) |
|  | Myocardial Infarction | 716 (0.5) | 1.09 (0.83,1.43) | 1.10 (0.84,1.44) | 1.08 (0.83,1.42) | 1.05 (0.78,1.41) | 1.04 (0.78,1.40) |
|  | Stroke | 3,278 (2.1) | 0.92 (0.82,1.05) | 0.65 (0.57,0.73) | 0.95 (0.84,1.08) | 0.96 (0.84,1.10) | 0.96 (0.84,1.10) |
|  | Heart Failure | 374 (0.2) | 0.60 (0.42,0.86) | 0.65 (0.45,0.93) | 0.65 (0.45,0.93) | 0.69 (0.47,1.04) | 0.70 (0.47,1.04) |

Abbreviations: PM_10_, particulate matter 10 micrometers or less in diameter. PM_2.5_, particulate matter 2.5 micrometers or less in diameter. PY, person-years.

^a^ Model 1: PM_10_ or PM_2.5_, sex, and age

^b^ Model 2: Model 1 + income, smoking, alcohol use, obese, and physical activity

^c^ Model 3: Model 2 + comorbidity of hypertension, diabetes, or hyperlipidemia

^d^ Model 4: Model 2 + area-level gross regional domestic products, percent of high school graduated or more, and percent of the elderly.

^e^ Model 5: Model 3 + area-level gross regional domestic products, percent of high school graduated or more, and percent of the elderly

**Table S3.** Adjusted hazard ratios and 95% confidence intervals of incident cardiovascular diseases including cardiovascular deaths for a 10 μg/m^3^ increase in long-term PM_10_ and PM_2.5_ concentrations for the previous 5 years by five health analysis models in 196,167 subjects of the National Health Insurance Service-National Sample Cohort 2.0 in South Korea for 2007-2015.

| Pollutant | Cardiovascular event | Number of events  (incidence per 10^3^ PY) | Hazard ratio (95% confidence interval) | | | | |
| --- | --- | --- | --- | --- | --- | --- | --- |
|  |  |  | Model 1^a^ | Model 2^b^ | Model 3^c^ | Model 4^d^ | Model5^e^ |
| PM_10_ | Total | 33,772 (21.4) | 0.97 (0.95,0.99) | 0.97 (0.95,0.99) | 0.96 (0.94,0.98) | 1.00 (0.98,1.03) | 1.00 (0.98,1.02) |
|  | Ischemic heart disease | 20,733 (13.1) | 1.01 (0.98,1.03) | 1.00 (0.98,1.03) | 1.00 (0.97,1.02) | 1.01 (0.98,1.04) | 1.00 (0.97,1.03) |
|  | Myocardial Infarction | 1,469 (0.9) | 0.95 (0.86,1.05) | 0.95 (0.86,1.04) | 0.94 (0.85,1.03) | 0.97 (0.87,1.09) | 0.97 (0.86,1.08) |
|  | Stroke | 10,235 (6.5) | 0.94 (0.90,0.97) | 0.94 (0.91,0.98) | 0.94 (0.91,0.97) | 1.01 (0.97,1.05) | 1.01 (0.97,1.05) |
|  | Heart Failure | 3,062 (1.9) | 0.84 (0.79,0.90) | 0.84 (0.79,0.90) | 0.84 (0.79,0.90) | 0.96 (0.89,1.04) | 0.96 (0.89,1.04) |
| PM_2.5_ | Total | 33,772 (21.4) | 1.00 (0.97,1.04) | 1.01 (0.97,1.04) | 1.01 (0.97,1.04) | 1.05 (1.00,1.09) | 1.05 (1.00,1.09) |
|  | Ischemic heart disease | 20,733 (13.1) | 1.10 (1.05,1.16) | 1.10 (1.05,1.16) | 1.10 (1.05,1.16) | 1.10 (1.04,1.16) | 1.10 (1.04,1.16) |
|  | Myocardial Infarction | 1,469 (0.9) | 1.07 (0.89,1.29) | 1.07 (0.89,1.29) | 1.07 (0.89,1.28) | 1.13 (0.92,1.38) | 1.13 (0.92,1.38) |
|  | Stroke | 10,235 (6.5) | 0.92 (0.86,0.99) | 0.71 (0.67,0.76) | 0.93 (0.87,1.00) | 1.01 (0.94,1.09) | 1.01 (0.94,1.09) |
|  | Heart Failure | 3,062 (1.9) | 0.70 (0.62,0.79) | 0.71 (0.63,0.81) | 0.71 (0.63,0.81) | 0.84 (0.74,0.97) | 0.84 (0.74,0.97) |

Abbreviation: PM_10_, particulate matter 10 micrometers or less in diameter. PM_2.5_, particulate matter 2.5 micrometers or less in diameter. PY, person-years.

^a^ Model 1: PM_10_ or PM_2.5_, sex, and age

^b^ Model 2: Model 1 + income, smoking, alcohol use, obese, and physical activity

^c^ Model 3: Model 2 + comorbidity of hypertension, diabetes, or hyperlipidemia

^d^ Model 4: Model 2 + area-level gross regional domestic products, percent of high school graduated or more, and percent of the elderly.

^e^ Model 5: Model 3 + area-level gross regional domestic products, percent of high school graduated or more, and percent of the elderly.

**Table S4**. Previous large cohort studies of the association between long-term exposure to PM_10_ or PM_2.5_ and incident cardiovascular diseases.

| Reference | Region | Nation | Name of  the cohort | Area | Population. | PM_10_/ PM_2.5_ | μg/m^3^ | Prediction  method | Address | Exposure window | Cox model |
| --- | --- | --- | --- | --- | --- | --- | --- | --- | --- | --- | --- |
| Present study | Asia | South Korea | NHIS-NSC 2.0 | Nationwide | Population- representative | All | 10 | Universal krigging | District | 1, 3, 5 yrs, 5 yrs before baseline | Fixed & time-dependent |
| Nishiwaki 2013 |  | Japan | JPHC study | 9 areas | Population-based | PM_10_ | 10 | Nearest monitor | Area | Whole period | Fixed |
| Liang 2020 |  | China | China-PAR | Nationwide | Population-based | PM^2.5^ | 10 | AOD-based | 1km^2^ grid | Whole period | Time-varying |
| Chen, 2005 | Northern America | The U.S. | AHSMOG | 3 MSAs | Non-Hispanic whites | All | 10 | Interpolation | Zip-code | 4 yrs | Time-dependent |
| Miller2007 |  |  | WHI | 36 MSAs | Women | All | 10 | Nearest monitor | 5-digit zip-code | 1 yr | Fixed |
| Puett 2008 |  |  | NHS | MSAs  (13 states) | Women | PM_10_ | 10 | GIS-based smoothing | Geo-coded | 1, 3, 12, 48- month | Time-varying |
| Puett 2009 |  |  | NHS | MSAs  (13 states) | Women | All | 10 | GIS-based smoothing | Geo-coded | 1, 3, 12, 48 months | Time-varying |
| Lipsett 2011 |  |  | CTS | California | Women | All | 10 | IDW interpolation | Geo-coded | 10 yrs | Fixed |
| Puett 2011 |  |  | HPFS | 13 states | Men | All | 4 | Spatiotemporal | Geo-coded | 12, 24, 36, 48 months | Time-varying |
| Hart 2015 |  |  | NHS | MSAs  (13 states) | Women | All | 10 | Spatiotemporal | Geo-coded | 12, 24, 60, 120 months | Time-varying |
| Loop 2018 |  |  | REGARDS | 8 states | Population-based | PM_2.5_ | 2.7 | AOD-based | Geo-coded | 1 yr | Fixed |
| Yadiz 2019 |  |  |  | 7 states | Medicare recipients | PM_2.5_ | 1 | Hybrid | 1km^2^ grid | Whole period | Fixed |
| Gan 2011 |  | Canada |  | Vancouver | Population-based | PM_2.5_ | 1.58 | LUR | Zip-code | 5 yrs | Fixed |
| Bai 2019 |  |  | ONPHEC | Ontario |  | PM_2.5_ | 3.5 | AOD-based | Zip-code | 3 yrs (MA) | Fixed |
| Atkinson 2013 | Europe | England |  |  | Patient only | PM_10_ | 3 | Air dispersion | Zip-code | 1 yr (MA) | Time- dependent |
| Stafoggia 2014 |  |  | ESCAPE |  |  | All | 10, 5 | LUR | Geo-coded | 4 yrs | Fixed |
| Cesaroni 2014 |  |  | ESCAPE |  |  | All | 10, 5 | LUR | Geo-coded | 4 yrs | Fixed |
| Downward 2018 |  | Netherlands | EPIC-NL | 4 cities |  | All | 10, 5 | LUR | Geo-coded | 1 yr | Fixed |

**Table S4**. Cont.

| Reference | Region | HR (95% CI) per increments in PM concentration | | | | | Effect modification |
| --- | --- | --- | --- | --- | --- | --- | --- |
|  |  | CVD | CHD or IHD | MI | Stroke | Heart failure |  |
| Present study | Asia | 1.00(0.98,1.02) / 1.04(1.00,1.09) | 1.00(0,97, 1.03) / 1.10(1.04,1.16) | 0.96(0.86,1.08) / 1.11(0.90,1.37) | 1.01(0.97,1.05) / 1.01(0.94,1.09) | 0.96 (0.89,1.04) / 0.84(0.73,0.96) | People with high-income, former smoker, non-obese people, diabetic patients |
| Nishiwaki 2013 |  | 0.76(0.67,0.87) / none | 1.20(0.95,1.64) / none | 1.37(1.03,1.82) / none | 0.68(0.59,0.78) / none |  | NR |
| Liang 2020 |  | 1.25(1.22,1.28) |  | 1.22(1.14,1.30) | 1.13(1.10,1.17) |  | Age, urbanicity, smoking |
| Chen, 2005 | Northern America |  | 1.22(1.01, 1.47) / 1.42(1.11,1.81)^a^ 0.94(0.82,1.08) / 0.90(0.76,1.05)^b^ |  |  |  | NR |
| Miller2007 |  | 1.04(0.97,1.10) / 1.24(1.09,1.41) | none / 1.21(1.04,1.42) | none / 1.06(0.85,1.34) | none / 1.28(1.02,1.61) |  | BMI, WH ratio, time to live in the current state |
| Puett 2008 |  |  | 1.10(0.94,1.29) / none | 0.94(0.77,1.15) / none^c^ |  |  | BMI of 30 or higher |
| Puett 2009 |  |  | 1.04(0.82,1.32) / 1.11(0.79,1.55) | 0.96(0.71,1.30) / 0.73(0.48,1.12)^c^ |  |  | Family history of MI, former smoker, the highest Quartile of median house value and family income |
| Lipsett 2011 |  |  |  | 0.98(0.83,1.16) / 0.98(0.83,1.16) | 1.06(1.00,1.13) / 1.14(0.99, 1.32) |  | NR |
| Puett 2011 |  | 1.03(0.95,1.10) / 0.99(0.90,1.10) | 1.04(0.92,1.17) / 0.97(0.83,1.13)^d^ | 1.00(0.87,1.14) / 1.06(0.90,1.25)^c^ | 1.22(0.98,1.52) / 0.76(0.58,0.99)^e^ 0.78(0.53,1.16) / 1.35(0.87,2.09)^f^ |  | Family history of MI, never- or current smoker |
| Hart 2015 |  | 1.05(0.96,1.14) / 1.11(0.96,1.29) | 1.02(0.91,1.15) / 1.05(0.86,1.29) |  | 1.07(0.95,1.20) / 1.18(0.96,1.45) |  | Diabetic patients, 70 years old or more |
| Loop 2018 |  |  |  | 0.85(0.73,0.99) / none^c^ |  |  | No effect modification |
| Yadiz 2019 |  |  |  | 1.03(1.02,1.03) | 1.03(1.03,1.03) | 1.05(1.05,1.05) | NR |
| Gan 2011 |  |  | none / 1.00(0.98,1.02) |  |  |  | More than 70 years old, higher neighborhood SES |
| Bai 2019 |  |  |  | none / 1.05(1.04,1.06)^g^ |  |  | Younger people, low-income residents |
| Atkinson 2013 | Europe |  |  | 0.98 (0.94, 1.01) / none | 0.98 (0.95, 1.01) / none | 1.06 (1.01, 1.11) / none | Lower deprivation index |
| Stafoggia 2014 |  |  |  |  | 1.11(0.90,1.36) / 1.19(0.88,1.62) |  | Rural area, never or former smoker |
| Cesaroni 2014 |  |  |  | 1.12 (1.01,1.25) / 1.13(0.98,1.30)^h^ |  |  | Aged 60 years or more |
| Downward 2018 |  | 1.20(0.96,1.50) / 0.98(0.75,1.28) | 1.14(0.85,1.53) / 0.80(0.55,1.15) | 1.27(0.77,2.09) / 0.83(0.44,1.57) |  | 2.09(0.99,4.40) / 0.44(0.16,1.20) | NR |

Abbreviations: HR, hazard ratio. CI, confidence interval. PM_10_, particulate matter 10 micrometers or less in diameter. PM_2.5_, particulate matter 2.5 micrometer or less in diameter. NHIS-NSC, National Health Insurance-National Sample Cohort; JPHC, Japan Public Health Center-based Prospective; China-PAR, Prediction for Atherosclerotic Cardiovascular Disease Risk in China; 33CCHS, 33 Communities Chinese Health Study; AHSMOG, Adventist Health Study on the Health Effects of Smog; WHI, Women’s Health Initiative; NHS, Nurses’ Health Study; CTS, California Teachers Study; HPFS, The Health Professionals Follow-up Study; REGARDS, REasons for Geographic And Racial Differences in Stroke; ONPHEC, Ontario Population Health and Environment Cohort; ESCAPE, European Study of cohorts for Air Pollution Effects; EPIC-NL, Dutch arm of the European Prospective Investigation into Cancer and Nutrition study; MSA, Metropolitan Statistical Area; GPRD, General Practice Research Database; PLS, partial least square; GIS, geographic information system; IDW, inverse distance weighted; LUR, land use regression; AOD, aerosol optical depth; GWR, Geographically Weighted Regression; MA, moving average; Cox PH model, Cox proportional hazard model; GLMM, generalized linear mixed model; CVD, cardiovascular disease; IHD, ischemic heart disease; MI, myocardial infarction; CHD, coronary heart disease; CHF, congestive heart failure; AMI, acute myocardial infarction; CVA, cerebrovascular disease; HR, hazard ratio; CI, confidence interval; NR, no report; BMI, body mass index; WH, waist-hip.

^a^only for females.

^b^only for males.

^c^hazard ratios and 95% confidence intervals for nonfatal myocardial infarction.

^d^hazard ratios and 95% confidence intervals for fatal coronary heart disease.

^e^hazard ratios and 95% confidence intervals for ischemic stroke.

^f^hazard ratios and 95% confidence intervals for hemorrhagic stroke.

^g^hazard ratios and 95% confidence intervals for acute myocardial infarction.

^h^hazard ratios and 95% confidence intervals for coronary events.

**Table S5.** Summary statistics of individual-level long-term concentration of PM_10_ and PM_2.5_ across four exposure periods and the number of events and incidence of total and subtypes of cardiovascular diseases in 196,167 subjects of the National Health Insurance Service-National Sample Cohort 2.0 in South Korea for 2007-2015 by the Seoul Metropolitan Area (SMA) and non-SMA.

| Pollutant/  Outcome | Category | Nationwide  (n=196,167) | SMA  (n=84,412) | Non-SMA  (n=38,162) |
| --- | --- | --- | --- | --- |
| PM_10_ | Previous 1 year | 50.5±6.8^a^ | 53.8±6.7 | 47.9±5.7 |
|  | Previous 3 years | 51.2±6.4 | 54.8±6.0 | 48.5±5.3 |
|  | Previous 5 years | 52.3±6.2 | 56.3±5.3 | 49.3±5.0 |
|  | 5 years before baseline | 55.7±6.6 | 61.4±2.8 | 51.5±5.4 |
| PM_2.5_ | Previous 1 year | 26.4±3.1 | 26.5±2.6 | 26.4±3.5 |
|  | Previous 3 years | 27.5±3.9 | 27.7±3.4 | 27.4±4.2 |
|  | Previous 5 years | 28.1±3.6 | 28.5±3.1 | 27.8±3.9 |
|  | 5 years before baseline | 31.2±3.4 | 32.3±1.8 | 30.4±4.1 |
| CVD | Total | 33,580 (21.3^b^) | 13,213 (18.3) | 20,367 (23.7) |
|  | IHD | 20,604 (13.1) | 8,502 (11.8) | 12,102 (14.1) |
|  | MI | 1,367 (0.9) | 535 (0.7) | 832 (1.0) |
|  | Stroke | 10,201 (6.5) | 3,788 (5.3) | 6,413 (7.5) |
|  | Heart failure | 3,033 (1.9) | 1,016 (1.4) | 2,017 (2.4) |

^a^ Mean± standard deviation

^b^ The number of events (the incidence) per 10^3^ person-year.

**Table S6.** Descriptive summary statistics of individual- and area-level characteristics, and long-term PM_10_ and PM_2.5_ concentrations of 196,167 total subjects of the National Health Insurance Service-National Sample Cohort 2.0 in South Korea for 2007-2015 by diagnosis and hospitalization of cardiovascular diseases.

| Characteristics |  | Total | Diagnosis | | Hospitalization | |
| --- | --- | --- | --- | --- | --- | --- |
|  |  |  | Censored | Case | Censored | Case |
| N |  | 196,167 | 162,587 | 33,580 | 190,036 | 6,131 |
| Sex (%) | Male | 53.5 | 54.0 | 51.4 | 53.3 | 59.8 |
| Average age  (years, mean±SD) |  | 46.6±11.0 | 45.1±10.4 | 53.5±11.2 | 46.3±10.9 | 55.4±11.7 |
| Age (years, %) | 30-64 | 92.1 | 94.3 | 81.2 | 92.6 | 74.1 |
|  | 65-80 | 8.0 | 5.7 | 18.8 | 7.4 | 25.9 |
| Income percentile (%)^a^ | 0 – 20% | 11.8 | 11.6 | 12.8 | 11.8 | 13.2 |
|  | 20 – 50% | 24.6 | 24.7 | 24.1 | 24.6 | 25.0 |
|  | 50 – 80% | 36.9 | 37.4 | 34.2 | 36.9 | 35.3 |
|  | 80 – 100% | 26.7 | 26.3 | 29.0 | 26.8 | 26.5 |
| Insurance type (%) | Self-employed | 40.3 | 39.5 | 44.1 | 40.2 | 43.7 |
|  | Employee | 59.7 | 60.5 | 55.9 | 59.8 | 56.3 |
| Smoking status (%) | Never | 67.7 | 67.2 | 70.1 | 67.8 | 63.2 |
|  | Former | 9.2 | 9.3 | 9.2 | 9.3 | 8.5 |
|  | Current | 23.1 | 23.6 | 20.8 | 22.9 | 28.3 |
| Alcohol use (%) | ≥ 3 times/week | 10.3 | 10.0 | 11.3 | 10.1 | 13.7 |
| Physical activity (%) | ≥ 3 times/week | 20.4 | 20.1 | 21.7 | 20.4 | 19.8 |
| BMI (%) | ≥ 25 kg/m^2^ | 33.4 | 32.1 | 39.5 | 33.3 | 37.5 |
| Comorbidity (%) | Hypertension^b^ | 21.0 | 18.9 | 31.1 | 20.6 | 34.2 |
|  | Diabetes^c^ | 6.4 | 5.6 | 10.3 | 6.2 | 13.4 |
|  | Hyperlipidemia^d^ | 12.0 | 11.4 | 15.3 | 11.9 | 16.1 |
| Region (%) | Metropolitan cities | 45.3 | 45.8 | 42.9 | 45.4 | 40.9 |
|  | Provinces | 54.7 | 54.2 | 57.1 | 54.6 | 59.1 |
| Area type (%) | Urban | 61.1 | 62.1 | 56.4 | 61.4 | 53.5 |
|  | Suburban | 24.1 | 24.0 | 24.8 | 24.0 | 26.0 |
|  | Rural | 14.8 | 13.9 | 18.8 | 14.6 | 20.6 |
| Gross Regional Domestic Products (%)^e^ | Very low | 8.0 | 7.4 | 11.0 | 7.9 | 12.5 |
|  | Low | 17.7 | 17.2 | 20.1 | 17.6 | 20.5 |
|  | High | 36.0 | 36.3 | 34.8 | 36.0 | 35.6 |
|  | Very High | 38.3 | 39.2 | 34.1 | 38.5 | 31.4 |
| Percent of the high school graduated or more (%)^f^ | Very low | 9.9 | 9.1 | 13.7 | 9.7 | 15.3 |
|  | Low | 15.9 | 13.4 | 18.0 | 15.8 | 18.4 |
|  | High | 34.4 | 34.9 | 31.7 | 43.4 | 32.7 |
|  | Very High | 39.9 | 40.6 | 36.6 | 40.1 | 33.7 |
| Percent of the elderly (%)^g^ | Very low | 37.6 | 38.8 | 32.1 | 37.9 | 30.1 |
|  | Low | 35.9 | 36.1 | 35.3 | 36.0 | 35.1 |
|  | High | 16.1 | 15.6 | 18.6 | 16.1 | 18.9 |
|  | Very High | 10.3 | 9.6 | 14.1 | 10.1 | 16.0 |
| Long-term PM exposure (mean±SD) | PM_10_ | 52.3±6.2 | 52.3±6.2 | 52.1±6.4 | 52.3±6.2 | 51.1±6.2 |
|  | PM_2.5_ | 28.1±3.6 | 28.1±3.6 | 28.2±3.7 | 28.1±3.6 | 27.7±3.6 |

Abbreviations: The data in the table was complete for all variables. SD, standard deviation; BMI, body mass index. SD, standard deviation.

^a^ NHIS-NSC provided income as percentiles.

^b^ Hypertension: diastolic blood pressure > 140 mmHg or systolic blood pressure > 90 mmHg.

^c^ Diabetes: fasting blood sugar level > 126 mg/dL.

^d^ Hyperlipidemia: total cholesterol level > 240 mg/dL.

^e^ Gross regional domestic products were categorized as very low (<1,110M USD), low (1,110 - 2,600M USD), high (2,600 - 8,354M USD), and very high (≥ 8,354M USD) based on the distribution across approximately 250 districts.

^f^ Percent of high school graduates was categorized as very low (< 34.3%), low (34.3 - 46.6%), high (46.6 - 53.2%), and very high (≥ 53.2%) based on the distribution across approximately 250 districts.

^g^ Percent of the elderly aged 65 years or more was categorized as very low (< 5.4%), low (5.4 - 8.2%), high (8.2 - 14.9%), and very high ( as ver based on the distribution across approximately 250 districts.

**Table S7.** Descriptive summary statistics of individual- and area-level characteristics, and long-term PM_10_ and PM_2.5_ concentrations of 196,167 total subjects of the National Health Insurance Service-National Sample Cohort 2.0 in South Korea for 2007-2015 by diagnosis and hospitalization of cardiovascular diseases.

| Characteristics |  | Total | Area type | | | |
| --- | --- | --- | --- | --- | --- | --- |
|  |  |  | Urban | Suburban | Rural |  |
| N (%) |  | 196,167 (100.0) | 119,980 (61.2) | 47,248 (24.1) | 289,39 (14.7) |  |
| Sex (%) | Male | 53.5 | 53.9 | 53.8 | 51.4 |  |
| Average age(years, mean±SD) |  | 46.6±11.0 | 45.6±10.4 | 46.8±11.4 | 50.1±12.2 |  |
| Age (years, %) | 30-64 | 92.1 | 94.4 | 90.7 | 84.7 |  |
| Income percentile (%)a | 0 – 20% | 11.8 | 11.6 | 12.2 | 12.0 |  |
|  | 20 – 50% | 24.6 | 24.0 | 24.5 | 27.3 |  |
|  | 50 – 80% | 36.9 | 36.1 | 37.7 | 38.6 |  |
|  | 80 – 100% | 26.7 | 28.3 | 25.7 | 22.1 |  |
| Insurance type (%) | Self-employed | 40.3 | 38.8 | 39.7 | 47.4 |  |
|  | Employee | 59.7 | 61.2 | 60.3 | 52.6 |  |
| Smoking status (%) | Never | 67.7 | 66.8 | 67.8 | 71.2 |  |
|  | Former | 9.2 | 9.5 | 9.5 | 7.6 |  |
|  | Current | 23.1 | 23.7 | 22.7 | 21.2 |  |
| Alcohol use (%) | ≥ 3 times/week | 10.3 | 9.9 | 10.1 | 12.0 |  |
| Physical activity (%) | ≥ 3 times/week | 20.4 | 21.9 | 19.1 | 16.1 |  |
| BMI (%) | ≥ 25 kg/m2 | 33.4 | 32.7 | 34.5 | 34.4 |  |
| Comorbidity (%) | Hypertensionb | 21.0 | 19.9 | 21.7 | 24.3 |  |
|  | Diabetesc | 6.4 | 6.0 | 6.9 | 7.3 |  |
|  | Hyperlipidemiad | 12.0 | 12.1 | 11.5 | 12.4 |  |
| Region (%) | Metropolitan cities | 45.3 | 70.7 | 0.0 | 13.8 |  |
|  | Provinces | 54.7 | 29.3 | 100.0 | 86.2 |  |
| Gross Regional Domestic Products (%)e | Very low | 8.0 | 0.5 | 1.9 | 49.3 |  |
|  | Low | 17.7 | 13.9 | 19.4 | 30.5 |  |
|  | High | 36.0 | 35.3 | 49.0 | 17.6 |  |
|  | Very High | 38.3 | 50.3 | 19.7 | 2.6 |  |
| Percent of the high school graduated or more (%)f | Very low | 9.9 | 0.0 | 6.7 | 56.0 |  |
|  | Low | 15.9 | 3.0 | 39.5 | 30.7 |  |
|  | High | 34.4 | 35.3 | 46.1 | 11.4 |  |
|  | Very High | 39.9 | 61.7 | 7.7 | 1.9 |  |
| Percent of the elderly (%)g | Very low | 37.6 | 56.0 | 8.5 | 8.9 |  |
|  | Low | 35.9 | 41.0 | 42.2 | 4.7 |  |
|  | High | 16.1 | 3.0 | 41.6 | 29.1 |  |
|  | Very High | 10.3 | 0.0 | 7.7 | 57.4 |  |
| Long-term PM exposure (mean±SD) | PM_10_ | 52.3±6.2 | 53.7±5.7 | 50.5±6.4 | 49.1±5.8 |  |
|  | PM_2.5_ | 28.1±3.6 | 28.9±3.5 | 26.7±3.2 | 26.8±3.6 |  |
| Cardiovascular events  (n, incidence per 10^3^ PY) | Total | 33,580 (21.3) | 18,949 (19.4) | 8,311 (22.0) | 6,320 (28.3) |  |
|  | Ischemic heart disease | 20,604 (13.1) | 12,352 (12.7) | 4,806 (12.7) | 3,446 (15.4) |  |
|  | Myocardial infarction | 1,367 (0.9) | 786 (0.8) | 308 (0.8) | 273 (1.2) |  |
|  | Stroke | 10,201 (6.5) | 5,290 (5.4) | 2,695 (7.1) | 2,216 (9.9) |  |
|  | Heart Failure | 3,033 (1.9) | 1,445 (1.5) | 866 (2.3) | 722 (3.2) |  |

Abbreviations: The data in the table was complete for all variables. SD, standard deviation; BMI, body mass index. SD, standard deviation.

^a^ NHIS-NSC provided income as percentiles.

^b^ Hypertension: diastolic blood pressure > 140 mmHg or systolic blood pressure > 90 mmHg.

^c^ Diabetes: fasting blood sugar level > 126 mg/dL.

^d^ Hyperlipidemia: total cholesterol level > 240 mg/dL.

^e^ Gross regional domestic products were categorized as very low (<1,110M USD), low (1,110 - 2,600M USD), high (2,600 - 8,354M USD), and very high (≥ 8,354M USD) based on the distribution across approximately 250 districts.

^f^ Percent of high school graduates was categorized as very low (< 34.3%), low (34.3 - 46.6%), high (46.6 - 53.2%), and very high (≥ 53.2%) based on the distribution across approximately 250 districts.

^g^ Percent of the elderly aged 65 years or more was categorized as very low (< 5.4%), low (5.4 - 8.2%), high (8.2 - 14.9%), and very high ( as ver based on the distribution across approximately 250 districts.

**
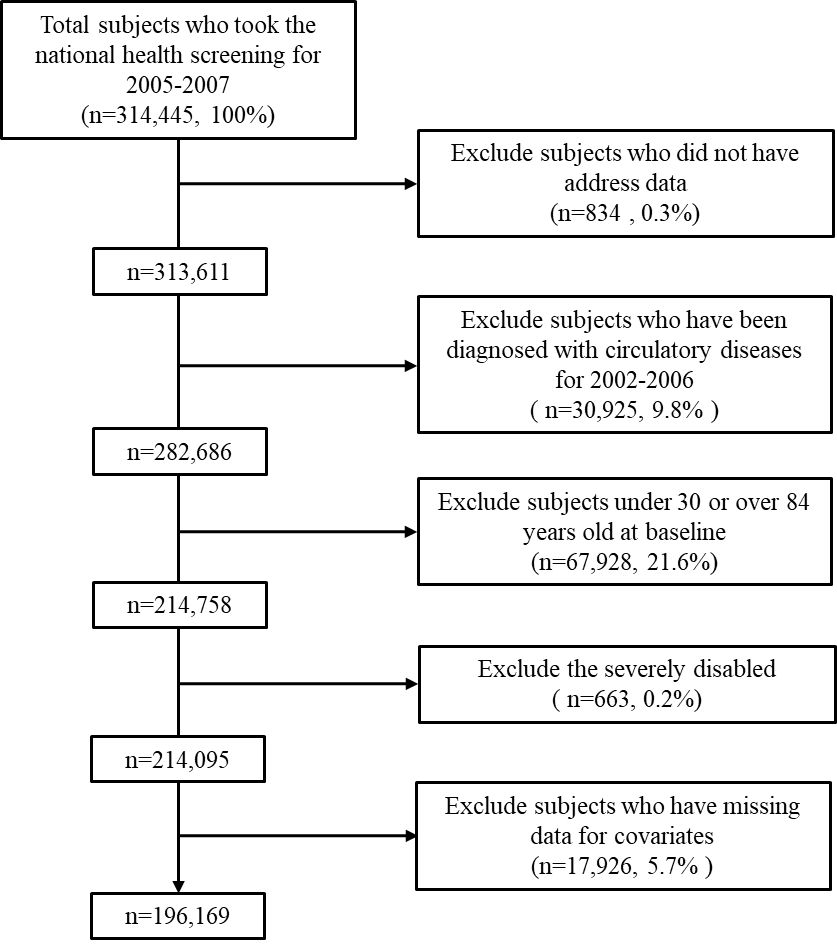
**

**Figure S1.** Schematic diagram of subject exclusion criteria and numbers of the National Health Insurance Service- National Sample Cohort 2.0 subjects included or excluded after the application of the criteria.


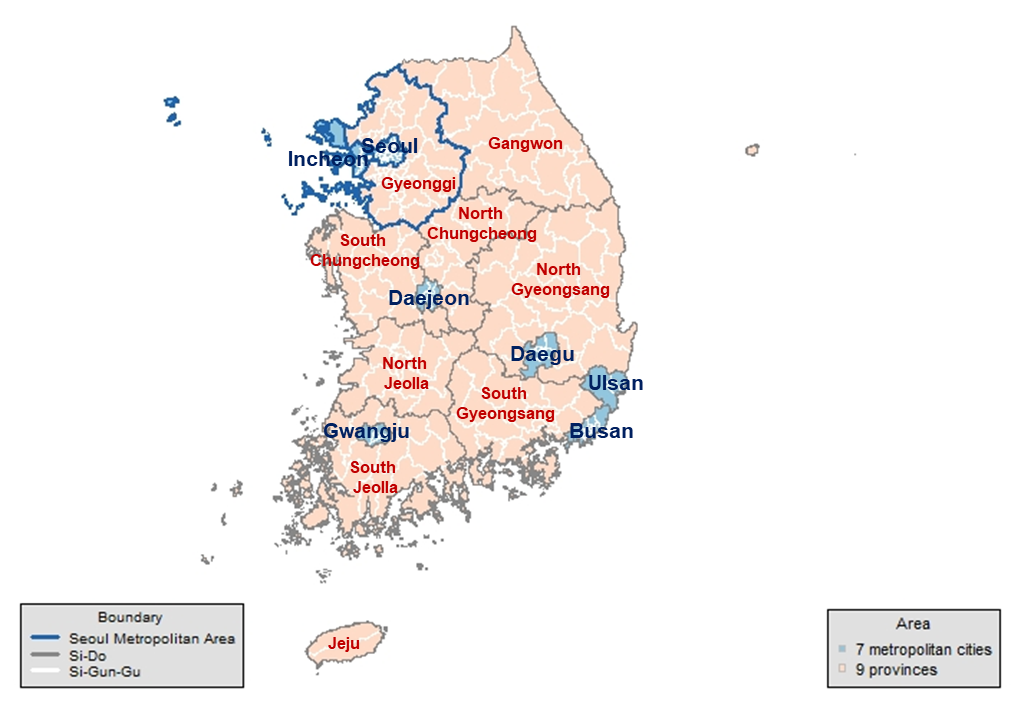


**Figure S2**. Map of 7 metropolitan cities and 9 provinces in South Korea, 2010.

**
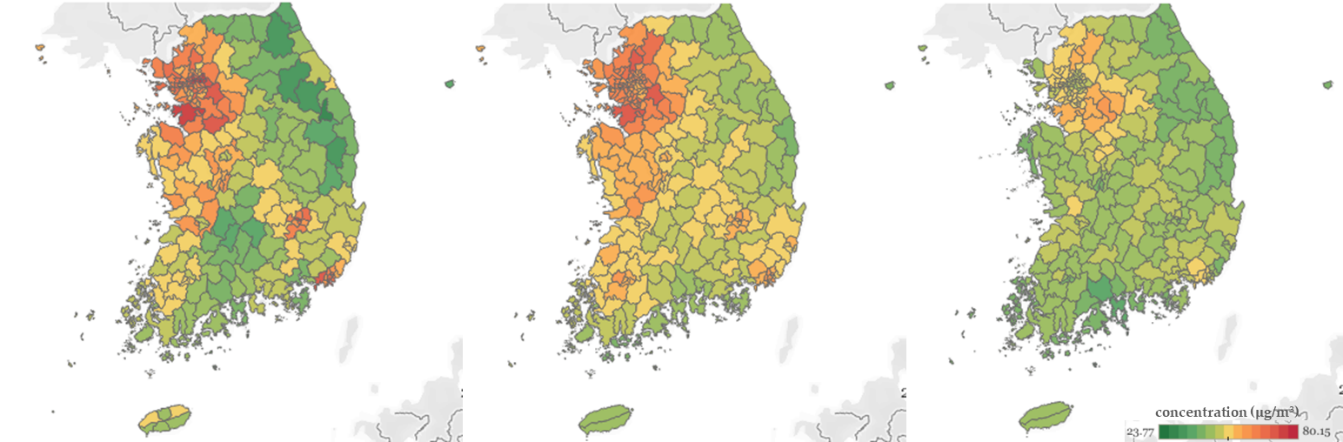
**

**Figure S3.** Maps of predicted annual-average concentrations of PM_10_ across 245,248, and 252 districts in South Korea by 2002, 2007, and 2014, respectively (modified from the maps on https://tabsoft.co/2T7v6ti).


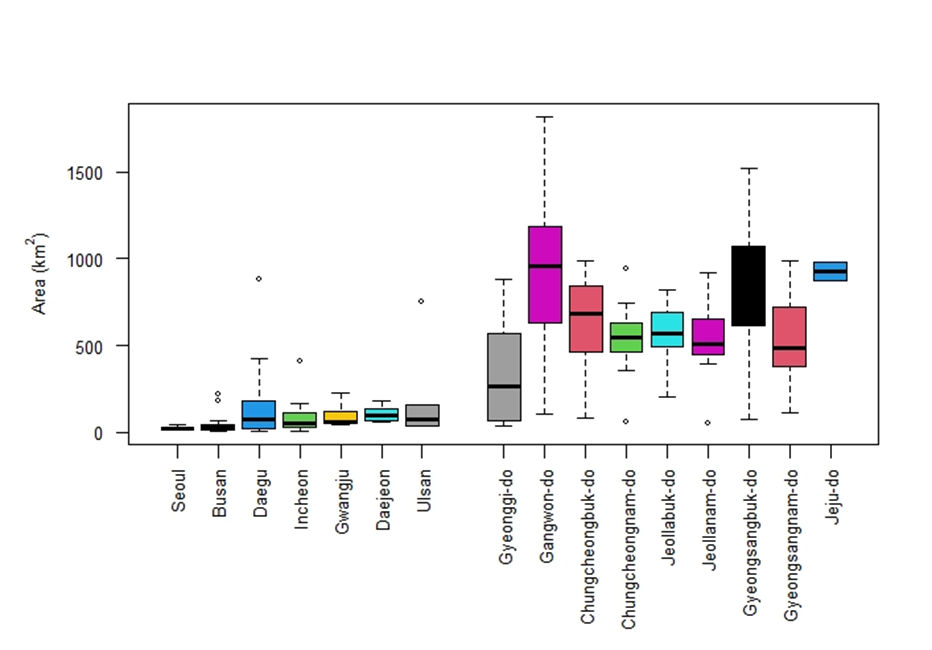


**Figure S4**. Box plot of the area sizes of districts by 7 metropolitan cities and 9 provinces, South Korea, in 2007.

**
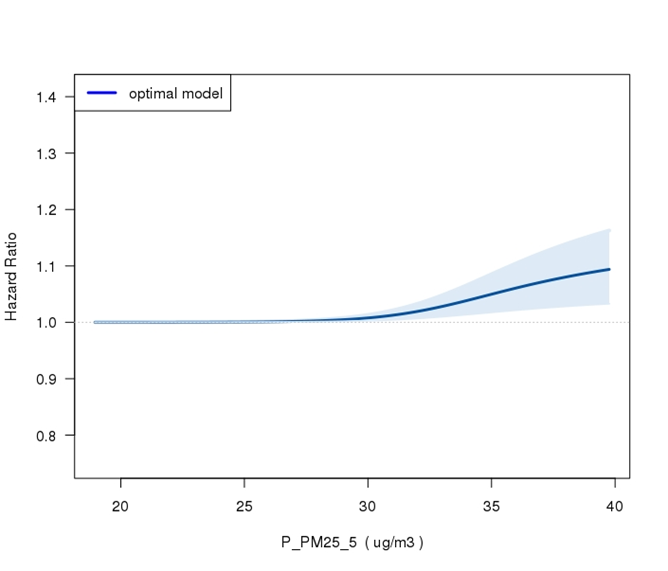
**

**Figure S5**. The concentration-response relationship between individual-level long-term concentration of PM_2.5_ for the previous 5 years and the incidence of total cardiovascular disease in 196,167 subjects of the National Health Insurance Service-National Sample Cohort 2.0 in South Korea for 2007-2015.

**
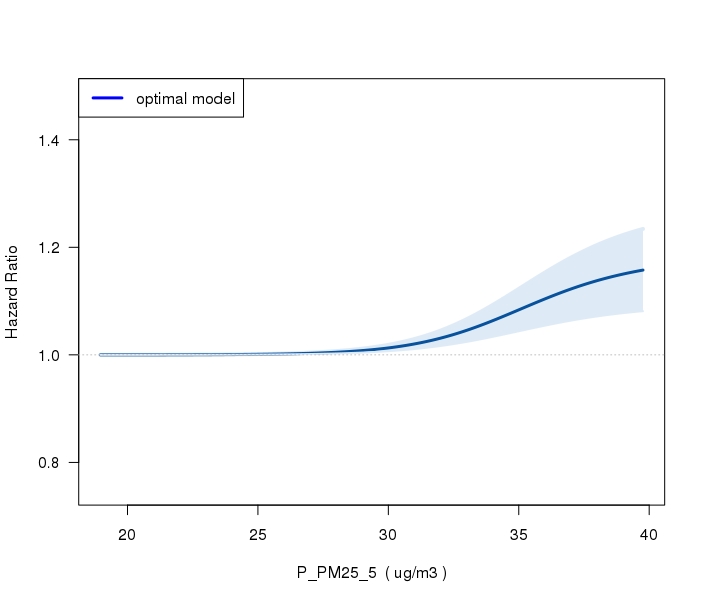
**

**Figure S6**. The concentration-response relationship between individual-level long-term PM_2.5_ concentration for the previous 5 years and the incidence of ischemic heart disease in 196,167 subjects of the National Health Insurance Service-National Sample Cohort 2.0 in South Korea for 2007-2015.

**
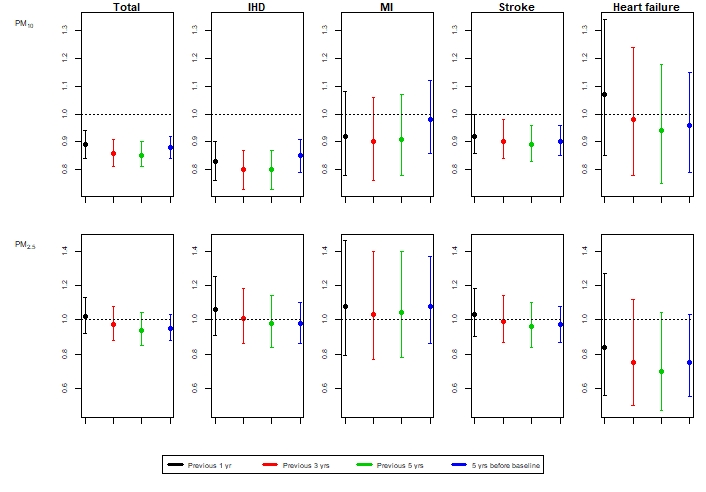
**

**Figure S7.** Adjusted hazard ratios and 95% confidence intervals of incidence of total cardiovascular diseases and four subtypes defined as hospital admissions for a 10 μg/m^3^ increase in long-term PM_10_ and PM_2.5_ concentrations by four exposure periods. Hazard ratios were adjusted for sex, age, income, smoking, alcohol use, obesity, physical activity, comorbidity (hypertension, diabetes, or hyperlipidemia), area-level gross regional domestic products, percent of high school graduated or more, and percent of the elderly. HR, hazard ratio; CI, confidence interval; PM_10_, particulate matter 10 micrometers or less in diameter; PM_2.5_, particulate matter 2.5 micrometers or less in diameter; IHD, ischemic heart disease; MI, myocardial infarction.

**
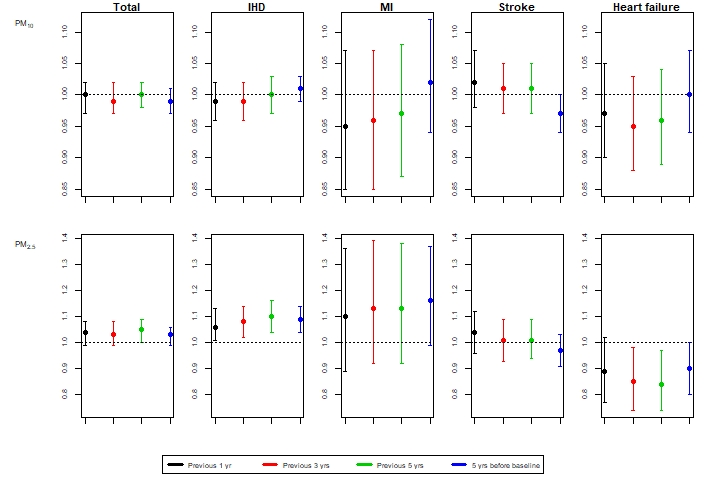
**

**Figure S8**. Adjusted hazard ratios and 95% confidence intervals of incident cardiovascular diseases including death from cardiovascular diseases for a 10 μg/m^3^ increase in long-term PM_10_ and PM_2.5_ concentrations by four exposure windows. Hazard ratios were adjusted for sex, age, income, smoking, alcohol use, obesity, physical activity, comorbidity (hypertension, diabetes, or hyperlipidemia), area-level gross regional domestic products, percent of high school graduated or more, and percent of the elderly. HR, hazard ratio; CI, confidence interval; PM_10_, particulate matter 10 micrometers or less in diameter; PM_2.5_, particulate matter 2.5 micrometers or less in diameter; IHD, ischemic heart disease; MI, myocardial infarction.
